# *Scutellaria baicalensis Georgi* in metabolic-associated fatty liver disease treatment: research progress

**DOI:** 10.3389/fphar.2025.1565461

**Published:** 2025-06-05

**Authors:** Liping Chen, Enhe Liu, Xi Zhao, Xiao Liu, Qin Dong, Yinpei Lou, Guoying Huang, Ling Li, Yuxin He

**Affiliations:** ^1^ School of Comprehensive Health Management, Xihua University, Chengdu, China; ^2^ School of Food and Bioengineering, Xihua University, Chengdu, China; ^3^ School of Pharmaceutical and Environmental Engineering, Sichuan Vocational College of Chemical Technology, Luzhou, China

**Keywords:** metabolic associated fatty liver disease, *Scutellaria baicalensis Georgi*, pharmacological activities, pharmacokinetics, production development

## Abstract

Metabolic associated fatty liver disease (MAFLD), previously known as nonalcoholic fatty liver disease (NAFLD), is a common liver condition marked by excessive fat accumulation exceeding 5% in the liver without significant alcohol consumption. It is closely linked to metabolic disorders such as obesity, type 2 diabetes, and dyslipidemia, with a rising global prevalence projected to escalate from 25% to 56% over the next decade. The pathogenesis of MAFLD is multifaceted, involving insulin resistance, inflammation, and oxidative stress, with progressive symptoms that can lead to severe liver conditions including non-alcoholic steatohepatitis (NASH). Current treatment options are limited, as established medications show variable efficacy and safety. *Scutellaria baicalensis Georgi* (*S. baicalensis*) a traditional Chinese herb rich in flavonoids, has garnered attention for its potential therapeutic effects on MAFLD. Its pharmacological activities, including anti-inflammatory, antioxidant, and lipid-regulating properties, position *S. baicalensis* as a promising candidate for MAFLD management. This article reviews the latest research progress of *S. baicalensis* in the treatment of MAFLD, explores its mechanism of action, pharmacokinetics, and the development of related products, aiming to clarify the pathogenesis of MAFLD and promote the development of new treatment and prevention strategies based on traditional Chinese medicine.

## 1 Introduction

**FIGURE 1:**
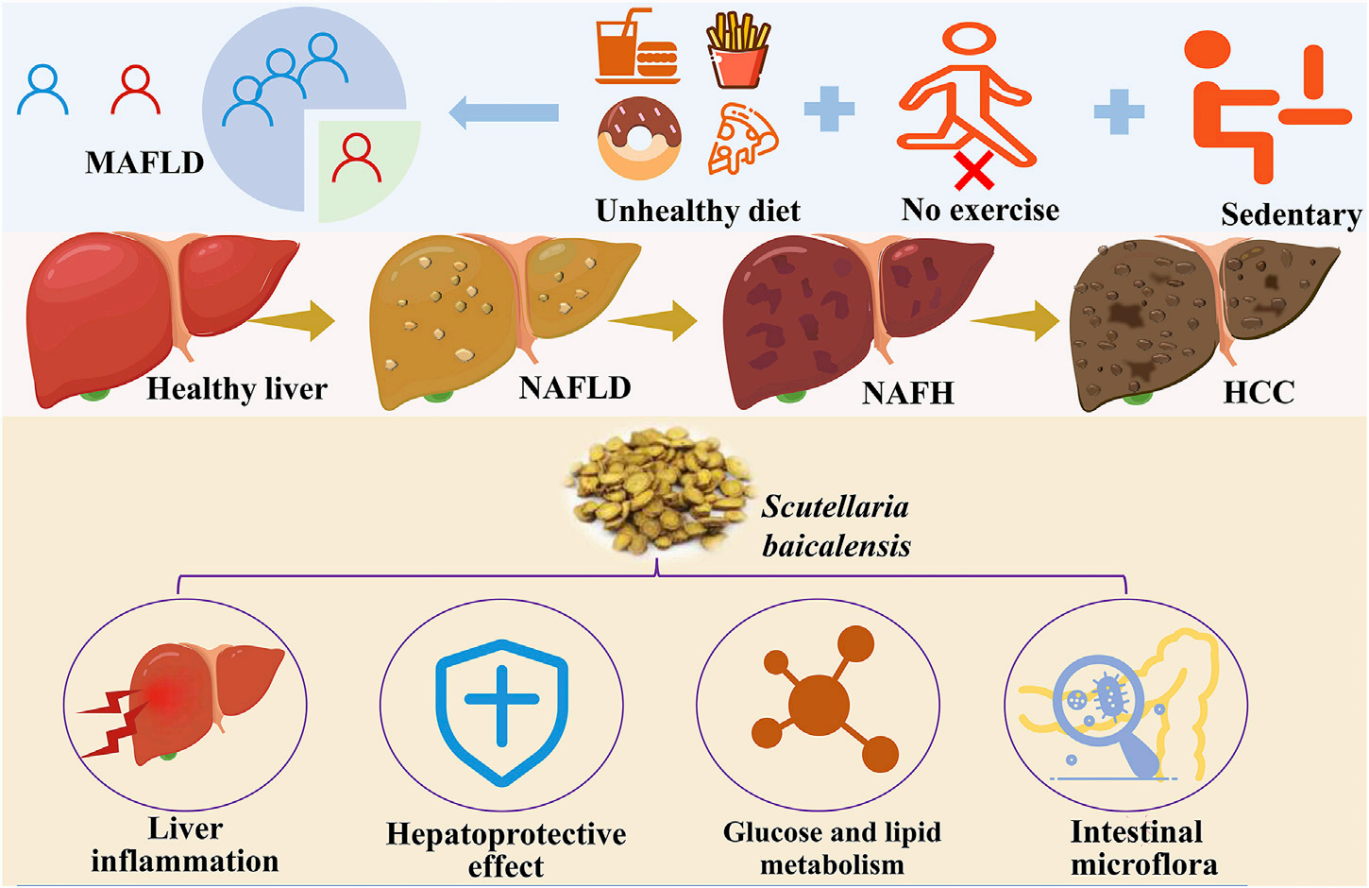
Epidemiology, pathogenesis, and potential protective methods of MAFLD (Created by iconfont. cn).

NAFLD is a common liver disease characterized by fat accumulation of more than 5% in the liver without excessive alcohol consumption. This disease is closely associated with metabolic syndromes and is commonly observed in individuals with obesity, type 2 diabetes, dyslipidemia, and hypertension (Palma et al., 2022; Zhao et al., 2018). The pathogenesis of NAFLD is complex and involves multiple factors such as insulin resistance, inflammatory responses, and oxidative stress. Clinically, NAFLD presents a wide range of symptoms, with many patients experiencing no noticeable signs in the early stages. As the disease progresses, symptoms like fatigue and discomfort in the right upper abdomen may occur. NAFLD can further advance to NASH, a more severe inflammatory condition of the liver that can lead to fibrosis, cirrhosis, and even hepatocellular carcinoma (Figure 1) (Ohtani et al., 2023). The global prevalence of NAFLD is rising annually, with projections indicating that prevalence will increase significantly from the current 25%–56% in China, the United States, and most European countries over the next decade (Huang et al., 2020). Lifestyle factors such as sedentary behavior and poor dietary habits are significant contributors to the development of NAFLD. Therefore, lifestyle interventions—including low-calorie diets, regular exercise, and weight management—are considered effective strategies for treatment and prevention (Elvira-Torales et al., 2019).

As NAFLD gains increasing attention globally, there is a growing recognition of the inherent limitations of the term “nonalcoholic.” This characterization places excessive emphasis on the absence of alcohol consumption while neglecting the significance of metabolic risk factors that drive the progression of NAFLD. The term NASH is even more complex, as “steatohepatitis” adds an additional layer of difficulty. In this context, diagnosing NASH not only requires the exclusion of alcohol intake but also histological confirmation of steatohepatitis. Studies have demonstrated significant inter- and intra-observer variability in the histological features, particularly ballooned hepatocytes, which are the main pathological hallmark. Given the lack of clarity regarding the relationship between NAFLD and metabolic risk factors, coupled with the overemphasis on alcohol, an international expert panel suggested in 2020 that the condition be renamed MAFLD. Consequently, this article will consistently use the term MAFLD.

Although several medications, such as metformin, vitamin E, and pioglitazone, are currently used to treat MAFLD (Guo et al., 2022), their efficacy and safety remain debated, and no specific drug for this condition has been established. Metformin has limited efficacy in reducing liver fat in the treatment of MAFLD (Goldberg et al., 2021), and long-term treatment may induce hepatotoxicity (Cone et al., 2010; Hashmi, 2011; Miralles-Linares et al., 2012). Vitamin E supplementation *notably raised* the *risk* of prostate cancer in healthy men (Klein et al., 2011). Pioglitazone is known to increase the risk of heart failure and fractures and might be associated with bladder cancer (Portillo-Sanchez et al., 2019; Azoulay et al., 2012; Violi and Cangemi, 2010). In summary, MAFLD is not only a significant concern for liver health but also poses a challenge to global public health. As its prevalence continues to rise, early detection and intervention will be key to improving patient outcomes and reducing the risk of associated complications. Thus, it is crucial to develop new treatment and prevention strategies, particularly those targeting metabolic abnormalities.

*S. baicalensis*, commonly known as Huangqin, is a perennial herbaceous plant belonging to the Lamiaceae family, primarily distributed in China and other East Asian regions (Jiang et al., 2017; Zhao Q. et al., 2016). The earliest documentation of *S. baicalensis* can be found in Shennong’s Classic of Materia Medica (Shennong Bencao Jing in Chinese), a classic Chinese herbal text written between 200 and 250 AD, which highlights its use for treating conditions related to bitterness, as well as lung and liver issues. The authoritative traditional Chinese medicine text “Bencao Gangmu” first published in 1,593, further reports *S. baicalensis*’s applications in treating diarrhea, dysentery, hypertension, hemorrhage, insomnia, inflammation, and respiratory infections (Chen et al., 2018; Orzechowska et al., 2020; Ji et al., 2015; Zhi et al., 2019; Yoon et al., 2020).

*S. baicalensis* is rich in flavonoids, including baicalin and baicalein, which contribute to its pharmacological activities (Hai-Juan et al., 2019; Chen et al., 2021). In recent years, there has been growing interest in *S. baicalensis*’s potential role in the treatment of MAFLD, a metabolic disorder characterized by the accumulation of fat in the liver (Chen et al., 2018; Liu et al., 2010; Sun et al., 2019). The pathogenesis of MAFLD is complex, involving factors such as inflammation, oxidative stress, and lipid metabolism dysregulation. Given *S. baicalensis*’s anti-inflammatory, antioxidant, and lipid-regulating properties, it is emerging as a promising candidate for MAFLD treatment research (Younossi et al., 2016; Semanticscholar, 2019; Yan et al., 2023; Na and sciences, 2019).

This review conducted a comprehensive literature search and screening of studies on *S. baicalensis* and its bioactive metabolites, such as baicalin and wogonin, in the treatment of MAFLD. Databases including PubMed, EMBASE, and Web of Science were systematically searched using combinations of the keywords “*S. baicalensis*” or its bioactive metabolites with “MAFLD” or “NAFLD.”The exclusion criteria were as follows: (I) Studies that did not investigate *S. baicalensis* or its bioactive metabolites for the treatment of MAFLD; (II) Studies in which *S. baicalensis* or its derivatives were not the primary intervention; (III) Studies focusing solely on the general pharmacological properties of *S. baicalensis* without specific relevance to MAFLD.

This review summarizes the current studies of *S. baicalensis* used for the treatment of MAFLD of pharmacological activity, mechanisms of action, pharmacokinetics and development of *S. baicalensis*-related products, to provide an important foundation for clarifying the pathogenesis of MAFLD and developing novel approaches for treatment and prevention based on Chinese herbal medicines.

## 2 The pathogenesis of MAFLD

MAFLD is widely recognized as a complex condition with multifactorial etiology, involving various contributors such as lipid metabolism disorders, endoplasmic reticulum (ER) stress, inflammatory activation, insulin resistance (IR), leptin resistance, oxidative stress, and dysbiosis of the gut microbiota (Rives et al., 2020; Wang et al., 2021; Wei et al., 2016). Among these, lipid metabolism disorders are central to the pathogenesis of MAFLD. Under normal circumstances, the liver regulates lipid synthesis and degradation through multiple mechanisms. However, in MAFLD patients, abnormal lipogenesis leads to significantly elevated levels of free fatty acids while fatty acid oxidation is markedly reduced (Metabolizm, 2012; Veracruz et al., 2021). This metabolic imbalance not only results in the accumulation of triglycerides (TG) in hepatocytes but also promotes the development of insulin resistance, subsequently triggering severe inflammatory responses (Hanley et al., 2004; Leung et al., 2008; Samuel et al., 2004; Singal et al., 2014; Szabo et al., 2015; Vozarova et al., 2002). Additionally, ER stress plays a critical role in the progression of MAFLD. When hepatocytes encounter excess lipids or other stressors, ER dysfunction can lead to the accumulation of unfolded proteins, initiating an ER stress response (Cullinan and Chemistry, 2004; Ajoolabady et al., 2023; Malhotra et al., 2008). This stress state activates multiple signaling pathways, resulting in inflammation and apoptosis of liver cells (Yu et al., 2019). Inflammatory responses are pivotal in the progression from MAFLD to NASH. Activated inflammatory cells in the liver, such as Kupffer cells and macrophages, release various pro-inflammatory cytokines, including tumor necrosis factor-alpha (TNF-α) and interleukin-6 (IL-6), which exacerbate hepatocyte damage and promote liver fibrosis (Kazankov et al., 2018).

Furthermore, insulin resistance and leptin resistance are significant metabolic features of MAFLD. Insulin resistance diminishes the liver’s response to insulin, disrupting glucose and lipid metabolism, while leptin resistance leads to energy imbalance, further exacerbating hepatic lipid accumulation. These metabolic abnormalities are closely linked to the onset of MAFLD and may contribute to disease progression. Oxidative stress represents another critical pathological mechanism; in MAFLD patients, levels of reactive oxygen species (ROS) are significantly elevated. Excess ROS can induce lipid peroxidation, protein damage, and DNA injury, exacerbating hepatocyte apoptosis and inflammatory responses. According to research by James P. Hardwick (Osborne et al., 2022), CYP4V2 may play a crucial role in regulating the progression of fat liver-related metabolic diseases. As simple steatosis progresses to hepatocellular carcinoma (HCC), ROS levels continue to rise, further underscoring the significance of oxidative stress in the advancement of MAFLD.

In the pathophysiology of MAFLD, the “two-hit” hypothesis is widely accepted. This theory posits that the progression of MAFLD occurs in two phases. The “first hit” refers to the accumulation of triglycerides within hepatocytes (i.e., steatosis) and the development of hepatic insulin resistance, which renders the liver more susceptible to subsequent damage. The “second hit” encompasses the secondary injuries resulting from the first hit, including alterations in the synthesis of lipotoxic factors, increased inflammation, oxidative stress, apoptosis, and liver fibrosis (Borrelli et al., 2018; Byrne and Hepatology, 2015; Haas et al., 2015; Rada et al., 2020). Moreover, ethnic and genetic differences significantly influence the occurrence of MAFLD. Research indicates that certain genetic variations may increase an individual’s susceptibility to MAFLD, while environmental factors, such as diet and lifestyle, can interact with genetic predispositions, further impacting the disease’s development (Caliceti et al., 2016).

As such, the “two-hit” hypothesis is often viewed by scientists as insufficient to explain all the molecular and metabolic abnormalities associated with MAFLD. In contrast, the “multiple-hit” theory suggests that the onset of MAFLD results from the combined effects of various damaging factors, including insulin resistance, adipokine secretion from adipose tissue, and the interplay between environmental (dietary) and genetic factors, such as epigenetics (Pierantonelli and Transplantation, 2019; Fang et al., 2018). The “multi-organ multiple impact” theory was proposed to explain the progression of MAFLD/NASH, arguing that free fatty acids, derived from lipolysis or *de novo* lipogenesis from dietary fats, exacerbate the burden on the liver. While fatty acids can be metabolized through β-oxidation and triglyceride degradation, saturation of these pathways leads to the formation of lipotoxic lipids, which may cause oxidative damage, ER stress, inflammation, and even cell death. Additionally, non-hepatic organs may directly or indirectly facilitate the development of NASH (Stefan et al., 2019; Kanwal et al., 2021; Long et al., 2022).

Recent studies have highlighted the potential protective effects of *S. baicalensis*, a traditional Chinese medicine, against MAFLD. Active metabolites in *S. baicalensis*, such as baicalin, have been shown to possess antioxidant, anti-inflammatory, and lipid metabolism-improving properties (Liu et al., 2020; Sun et al., 2019; Yang et al., 2020; Shi et al., 2020; Na and sciences, 2019). Research indicates that Scutellaria can reduce fat accumulation in hepatocytes and suppress inflammatory responses by regulating the expression of lipid metabolism-related genes (Shi et al., 2020; Geethangili et al., 2021). Furthermore, Scutellaria may promote gut health by improving the composition of the gut microbiota, thereby indirectly alleviating the symptoms of MAFLD (Ansari et al., 2020). Therefore, *S. baicalensis* can treat numerous pathophysiological mechanisms associated with NAFLD to ameliorate it.

## 3 The therapeutic mechanism of *S. baicalensis* for MAFLD

According to a comprehensive analysis of the literature, bioactive substances from *S. baicalensis*, including baicalin and baicalein, have been demonstrated antioxidant and anti-inflammatory qualities (Zhong and Liu, 2018; Xin et al., 2014), thereby alleviating liver damage and improving the pathological state of MAFLD. The therapeutic effects of *S. baicalensis* on MAFLD also involved other pathophysiological processes, including the improvement of insulin resistance, regulation of dyslipidemia, and modulation of the gut microbiota.

It has been suggested that *S. baicalensis* improves insulin sensitivity by modulating signaling pathways such as insulin signaling, thereby ameliorating disorders of glucose and lipid metabolism (Noh et al., 2021). In terms of regulating dyslipidemia, *S. baicalensis* has been found to reduce serum levels of total cholesterol, triglycerides, and other lipids through various mechanisms (Yan et al., 2022). Furthermore, during the combined treatment of MAFLD with other drugs, *S. baicalensis* has been shown to enhance therapeutic efficacy through its multitarget actions. These findings have demonstrate that *S. baicalensis* could treat or improve MAFLD through multiple pathways, and the specific mechanisms will be discussed in the following sections.

### 3.1 *S. baicalensis* regulates liver inflammation in the treatment of MAFLD

The development of MAFLD is closely associated with chronic inflammation caused by an imbalance between anti-inflammatory and pro-inflammatory markers. Extensive experimental studies have shown that *S. baicalensis* could alleviate hepatic inflammation by blocking the NF-κB signaling pathway, thus treating or delaying the onset of MAFLD. Furthermore, *S. baicalensis* treats MAFLD by controlling hepatic inflammation, altering macrophage polarization (Junior et al., 2021), and exhibiting antioxidant effects through the regulation of Nrf2 transcription factors and other mechanisms (Xin et al., 2014).

In multiple *in vitro* and *in vivo* studies, it has been well established that *S. baicalensis* exhibits potent anti-inflammatory effects and is demonstrated as a potential anti-inflammatory agent that plays an important role in reducing the inflammatory response in MAFLD. Therefore, we will next review the therapeutic effects of *S. baicalensis* on MAFLD inflammation, focusing on three key aspects: the NF-κB signaling pathway, macrophages, and oxidative stress.

#### 3.1.1 The NF-κB signaling pathway

The activation of NLRP3 has plays a critical role in the pathogenesis of MAFLD (Zou et al., 2022). Numerous studies have demonstrated that the NF-κB-mediated signaling pathway increases NLRP3 expression, which subsequently elevates the expression levels of inflammatory factors such as Pro-IL-1β, TNF-α, and IL-6. The NLRP3 inflammasome is composed of three proteins: NLRP3, ASC, and pro-caspase-1 (Shao et al., 2015). Upon activation of NLRP3, pro-caspase-1 undergones autolytic cleavage and cleaves the precursors of inflammatory cytokines, activating their inflammatory activity. It also cleaves GSDMD, releasing its N-terminal fragment, GSDMD-N, which forms pores on the cell membrane, resulting in membrane rupture and pyroptotic cell death (Fu et al., 2024). This process causes fibrosis in normal hepatocytes and accelerates the progression of MAFLD.

While treating MAFLD rats with *S. baicalensis*, researchers have discovered through molecular docking techniques that baicalin exhibited high affinity for TLR4 and NF-κB. This finding suggests that baicalin might alleviate inflammation and treat MAFLD by forming a robust hydrogen bond network with various amino acid residues of TLR4 and NF-κB, thereby blocking the NF-κB signaling pathway (Yan et al., 2023). Another study demonstrated the antifibrotic effects of baicalin in SD rats with thioacetamide-induced cirrhosis, revealing that its mechanism involved inhibition of NLPR3 and NF-κB signaling pathways, thereby reducing the production of inflammatory cytokines (Zaghloul et al., 2022). Gao et al. established a mouse model of MAFLD using a high-fat diet (HFD) and treated the mice with baicalin. They have found that intervention with baicalin inhibited the activity of NF-κB, reduced its nuclear translocation, and downregulated the expression of inflammation-related genes, thereby alleviating hepatic inflammatory responses. Additionally, activation of the Nrf2 signaling pathway and inhibition of SREBP1 together improved overall liver function (Gao et al., 2023). In summary, baicalin has demonstrated significant anti-inflammatory and anti-fibrotic effects by blocking NF-κB activation and inhibiting NLRP3, thereby reducing the production of inflammatory factors and improving hepatic metabolic function, providing new insights for the treatment of MAFLD.

#### 3.1.2 Macrophages

Based on their functional status, macrophages are classified into pro-inflammatory M1 macrophages and anti-inflammatory M2 macrophages. M1 macrophages induce robust inflammatory responses by releasing pro-inflammatory cytokines such as IL-1β, IL-6, IL-8, IL-12, and TNF-α, but excessive activation resultes in tissue damage. In contrast, M2 macrophages exhibite anti-inflammatory and reparative functions, primarily through the secretion of Arg-1, IL-10, TGF-β, and matrix metalloproteinases, contributing to tissue repair, wound healing and cellular debris clearance (Arabpour et al., 2021; Gordon and Martinez, 2010). In the treatment of MAFLD, M1 macrophages have been identified as primary effector cells responsible for excessive hepatic inflammatory responses (Junior et al., 2021). A study demonstrated that baicalin could repolarize lipopolysaccharide (LPS)-induced M1 macrophages into an M2 phenotype characterized by the downregulation of IRF5, TNF-α, and IL-23 (Zhu et al., 2016). Furthermore, subsequent research revealed that baicalin could induce the differentiation of macrophages into the anti-inflammatory M2c subtype and significantly enhance the expression and secretion of anti-inflammatory factors such as IL-10, IL-13 and IL-4 by activating the MERTK signaling pathway and suppressing it at the same time pro-inflammatory cytokines such as IL-12 and IFN-γ. This mechanism not only promoted immune tolerance and tissue repair but also improved the pathological symptoms of diet-induced MAFLD in murine models by enhancing hepatic HDL production and circulation, improving triglyceride transport, and inhibiting fatty acid synthesis (Junior et al., 2021).

Researchers have discovered that baicalin inhibits pan-apoptosis in Kupffer cells in a mouse model of hemophagocytic lymphohistiocytosis, thereby alleviating hepatic inflammation and organ damage (You et al., 2024). Similarly, Liu et al. demonstrated that baicalin treatment in a choline-deficient diet-induced mouse model of MAFLD significantly reduced macrophage infiltration and reversed hepatic lipid accumulation by suppressing the TLR4 signaling cascade. This effect protected the mice from MAFLD progression and inhibited further disease-related fibrosis (Liu et al., 2020). In another study, it was found that baicalin significantly decreased Ly6C^hi^ monocytes, M1 adipose tissue macrophages (ATMs), and M1 Kupffer cells in HFD-induced obese mice. Conversely, it increased anti-inflammatory M2 ATMs, hepatic CD4^+^ T cells, and the CD4/CD8 ratio (Noh et al., 2021). The study demonstrated that CD4^+^ T cells suppressed inflammatory responses by regulating the function of ATM, whereas CD8^+^ T cells promoted the polarization of M1 macrophages. Consequently, increasing the CD4/CD8 ratio enhances immune surveillance, inhibited inflammation, and significantly improved obesity by modulating glucose and lipid metabolism, thereby preventing further hepatic fat accumulation (Conroy et al., 2016; Nishimura et al., 2009). In summary, baicalin has exhibited substantial potential in the treatment of MAFLD by regulating macrophage polarization and inflammatory responses.

#### 3.1.3 Regulating inflammation through oxidative stress

**FIGURE 2:**
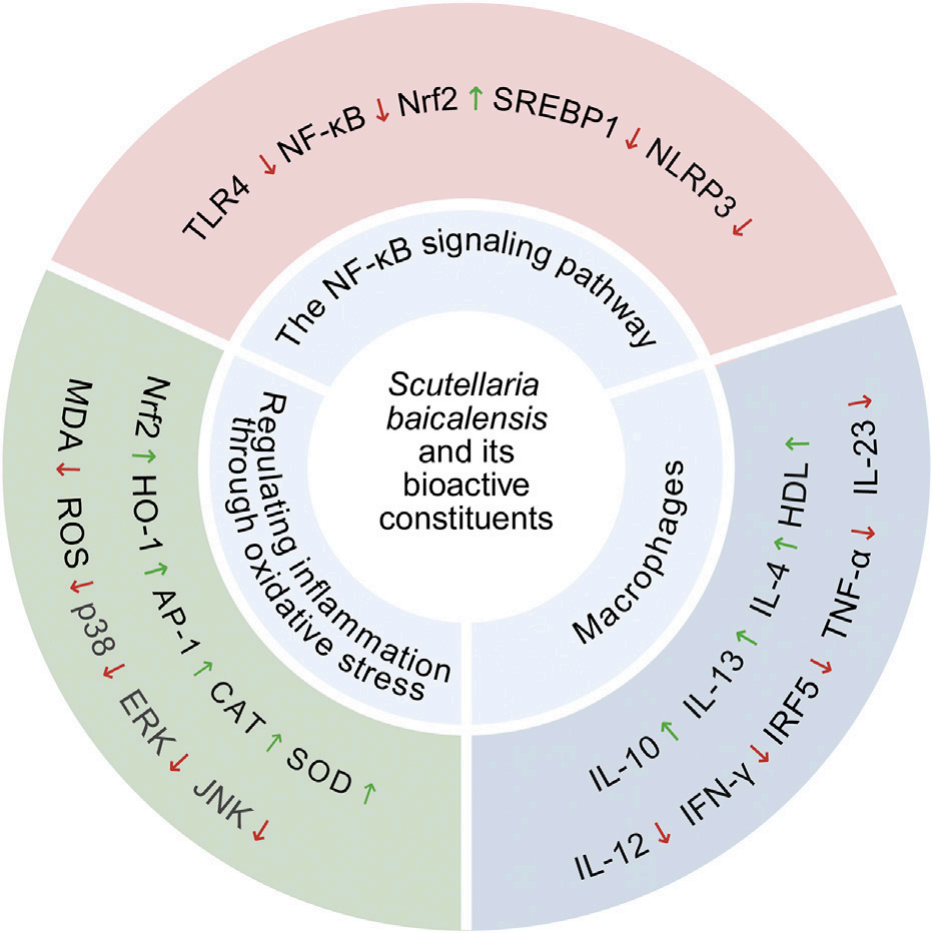
*S. baicalensis* and its bioactive metabolites regulates molecular targets related to oxidative stress and inflammation, including enzymes, proteins, chemical molecules, and inflammatory factors and Anti-inflammatory factor.

Oxidative stress plays a critical role in the pathological states of the liver (Tauil et al., 2024). Park et al. used a LPS-induced liver injury mouse model and found that heat-treated *S. baicalensis* significantly inhibited reactive ROS level in both serum and liver. The mechanism underlying this effect was likely associated with the suppression of oxidative stress-mediated activation of MAPK (p38, ERK and JNK), NF-κB and AP-1, which effectively ameliorated liver damage in LPS-treated mice (Park et al., 2017). Zhong et al. found that in diet-induced NASH mice, baicalin treatment alleviated oxidative stress damage in liver tissue by reducing MDA levels and significantly enhancing the activity of SOD (Zhong and Liu, 2018). Further studies showed that baicalin or baicalein significantly reduced oxidative stress in MAFLD mice and NASH rats by upregulating the Nrf2/HO-1 signaling pathway in the liver and increasing the activities of SOD and catalase, thereby protecting the liver from ROS -induced damage was protected and preventing further damage (Xin et al., 2014; Liu et al., 2023). In addition, Gao et al. by applying baicalin in a tissue-engineered MAFLD liver model, observed that it significantly alleviated oxidative stress in the model through various mechanisms, including reducing ROS levels, enhancing the expression and activity of antioxidant enzymes, and mitigating cell apoptosis (Gao et al., 2022). Taken together, the therapeutic effects of baicalin and its active metabolites in the treatment of MAFLD may be achieved primarily through antioxidant stress mechanisms (Figure 2).

### 3.2 *S. baicalensis* delays MAFLD through its hepatoprotective effect

Multiple studies have shown that *S. baicalensis* exerts significant hepatoprotective effects, with its mechanisms varying depending on the type of liver injury. LPS stimulation induces the release of TNF-α, IL-6, and IL-1β, triggering liver damage and potentially progressing to MAFLD (Laveti et al., 2013). Studies have shown that baicalin significantly reduces the release of inflammatory factors such as TNF-α by downregulating TLR4 expression and inhibiting NF-κB activation, thereby exerting protective effects against LPS-induced liver damage (Cheng et al., 2017).

Researchers have used carbon tetrachloride to induce liver damage to establish a liver fibrosis model. The studies have shown that baicalin can could reduce the serum activities of alanine aminotransferase (ALT), aspartate aminotransferasev (AST) and alkaline phosphatase (ALP), and improve liver damage caused by carbon tetrachloride (CCl_4_) (Qiao et al., 2011). Baicalin magnesium, a water-soluble compound isolated from *S. baicalensis* aqueous solution, significantly improves liver injury, lipid deposition, inflammatory response, and oxidative stress in a high-fat diet-induced NASH model, thereby providing protective effects on the liver (Guan et al., 2023).

In drug-induced liver injury, *S. baicalensis* has also shown significant preventive and curative effects. In a mouse model of acetaminophen (APAP)-induced liver injury, baicalin treatment was effective in reducing the levels of TNF-α, IL-6, IL-17, and MPO, thereby achieving a protective effect on the liver (Liao et al., 2016). Another study demonstrated that baicalein and baicalin alleviated APAP-induced liver injury by activating the Nrf2 antioxidant pathway. Molecular docking results indicated that baicalein and baicalin might block the interaction between Nrf2 and Keap1, inducing Nrf2 phosphorylation to activate Nrf2, thereby exerting antioxidant effects through its downstream proteins to reduce APAP-induced hepatotoxicity (Shi et al., 2018). Zhao et al. discovered that exosomes derived from mesenchymal stem cells pretreated with baicalin successfully alleviated hepatocyte ferroptosis following acute liver injury. The main mechanism was that baicalin upregulated the expression of P62, which regulated hepatocyte iron homeostasis through activation of the Keap1-NRF2 pathway (Zhao et al., 2022).

**FIGURE 3:**
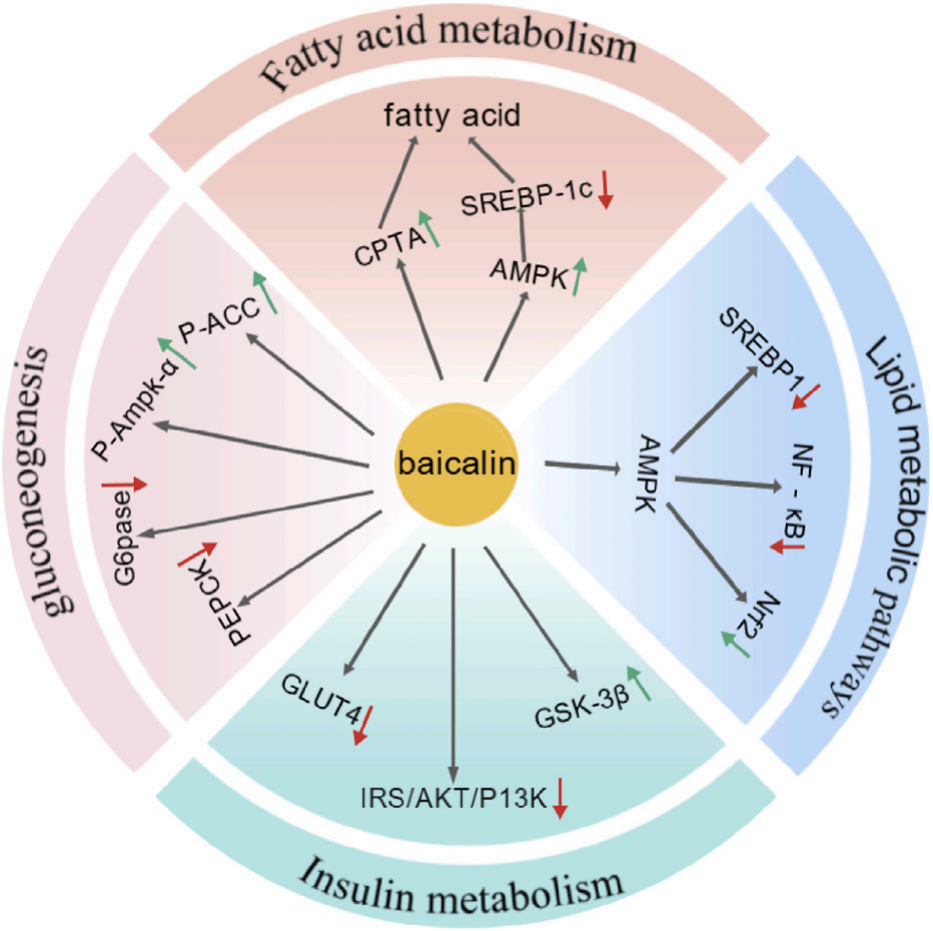
*S. baicalensis* and its active metabolites had exhibited protective effects against various liver injuries by reducing inflammatory factors, activating antioxidant pathways, and regulating related pathways (Created by BioGDP.com).

Taken together, *S. baicalensis* exhibites protective effect against various types of liver injury. In LPS-induced inflammation-related liver injury, baicalin inhibited the TLR4-NF-κB pathway to exert its effect. In liver injury models induced by CCl_4_ and HFD, metabolites of *S. baicalensis* improved several biomarkers. In case of drug-induced liver injury, *S. baicalensis* alleviated the damage through multiple mechanisms, such as the reduction of inflammatory factors, the activation of antioxidant pathways and the regulation of relevant signaling pathways (Figure 3).

### 3.3 S. baicalensis is treates MAFLD by improving glucose and lipid metabolism

The key bioactive compounds in *S. baicalensis* significantly improve the symptoms of MAFLD by regulating lipid and glucose metabolism. Multiple mechanisms are involved in improving glucose and lipid metabolism. In terms of lipid metabolism, they regulate fatty acid metabolism, promote fatty acid oxidation, and reduce fatty acid synthesis (Li et al., 2022b). In terms of glucose metabolism, the primary mechanism involved modulation of insulin signaling pathways and inhibition of gluconeogenesis-related pathways, thereby enhancing insulin sensitivity and decreasing glucose production in the liver (Wang et al., 2017).

#### 3.3.1 The lipid metabolism

Baicalin and baicalein have been shown to regulate lipid metabolism by modulating the pathways involved in fatty acid metabolism, lipid synthesis, and fat breakdown, thereby reducing hepatic fat accumulation. The study found that baicalein inhibited SREBP1c/ChREBP, activated AMPK and PPARα signaling pathways, and modulate the processes of fatty acid synthesis, elongation, and oxidation, thereby exerting a preventive effect against liver steatosis caused by fructose consumption in rats (Li et al., 2022b). Dai et al., through chemoproteomics analysis, have identified CPT1A as one of the critical targets within carnitine palmitoyltransferase 1 (CPT1), which serves as a rate-limiting enzyme in mitochondrial fatty acid β-oxidation. Treatment with baicalin resulted in significant reductions in body weight, liver weight and blood lipid levels in mice fed a high-fat diet. Overall, baicalin was found to bind CPT1A, activate its activity, and accelerate fatty acid degradation, thereby significantly alleviating nutritional hepatic steatosis-related symptoms. (Dai et al., 2018).

In addressing alcohol-induced hepatic steatosis, baicalin was able to ameliorate alcohol-induced pathological changes both *in vivo* and *in vitro*. This effect was achieved by activating lipolysis through the regulation of competitive binding between PNPLA3 and ATGL, mediated by SREBP1c (Li et al., 2022a). In non-alcoholic hepatic steatosis, baicalin improves high-fat diet-induced MAFLD by inhibiting SREBP1 and NF-κB signaling pathways via AMPK-mediated mechanisms and activating the Nrf2 signaling pathway (Gao et al., 2023). The activation of AMPK had attenuates the proteolytic processing of SREBP-1c, suppressed lipogenesis, and stimulated fatty acid oxidation, ultimately leading to reduced fat accumulation in the liver (Fang et al., 2018). Moreover, Guo et al. conducted an experiment in which male rats were divided into three groups: normal diet, high-fat diet, and high-fat diet with long-term baicalin administration. Their results demonstrated that prolonged baicalin treatment significantly reduced body weight, liver weight, and blood lipid levels in high-fat diet-induced obese rats (Guo et al., 2009).

#### 3.3.2 The glucose metabolism

Insulin resistance is a key feature of MAFLD, and studies have demonstrated that *S. baicalensis* and its active metabolites improve MAFLD treatment by regulating glucose metabolism. Multiple studies have shown that baicalin activates the insulin signaling pathway and enhances insulin sensitivity. As key regulators of glycogen synthesis, AKT/GSK-3β have been implicated in these processes. In HepG-2 cells with insulin resistance, Wang et al. found that baicalin suppressed gluconeogenesis by activating AMPK or AKT (Wang et al., 2017). Using a similar model, Miao et al. observed in insulin-resistant HepG-2 cells and prediabetic mice that baicalin prevented downregulation of the IRS/PI3K/AKT signaling pathway, reducing GLUT4 expression, and mitigating enhanced GSK-3β activity, thereby improving dyslipidemia and hyperglycemia in obese mice (Miao et al., 2024). Furthermore, Xi et al. revealed that baicalin activated the AKT signaling pathway and inhibited GSK3β activity, elucidating the mechanism by which baicalin enhanced insulin sensitivity and reduced ectopic fat storage (Xi et al., 2016). Taken together, baicalin alleviates insulin resistance and regulates hepatic glucose metabolism by activating the insulin signaling pathway.

Additionally, baicalin can reduce glucose production by inhibiting the expression of genes related to gluconeogenesis. A HFD induced insulin resistance and ectopic fat storage in skeletal muscle, which is a key feature of metabolic syndrome (Xi et al., 2016). Using high-fat diet-induced obese mice as a model, Fang et al. found that baicalin inhibited the phosphorylation of p38 MAPK, which in turn suppressed the expression and activity of PGC-1α.Acetyl-CoA carboxylase (ACC) is was a key enzyme in fatty acid synthesis, and baicalin alleviated the inhibitory effects of high-fat diet-induced phosphorylation of skeletal muscle ACC and AMP-activated protein kinase (AMPKα) (Xi et al., 2016). Phosphoenolpyruvate carboxykinase (PEPCK) and glucose-6-phosphatase (G6Pase) are key enzymes in hepatic gluconeogenesis, and their activity directly impactes blood glucose levels. Baicalin reduced the expression of these key enzymes, thereby decreasing fatty acid synthesis and storage (Fang et al., 2019). Using a high-fat diet-induced obese mouse model, Fang et al. found that baicalin inhibited the phosphorylation of p38 MAPK, which in turn suppressed the expression and activity of PGC-1α. This led to a reduction in the expression and activity of gluconeogenesis-related enzymes, significantly improving hepatic insulin resistance and gluconeogenic activity in high-fat diet-induced obese mice (Fang et al., 2019).

**FIGURE 4:**
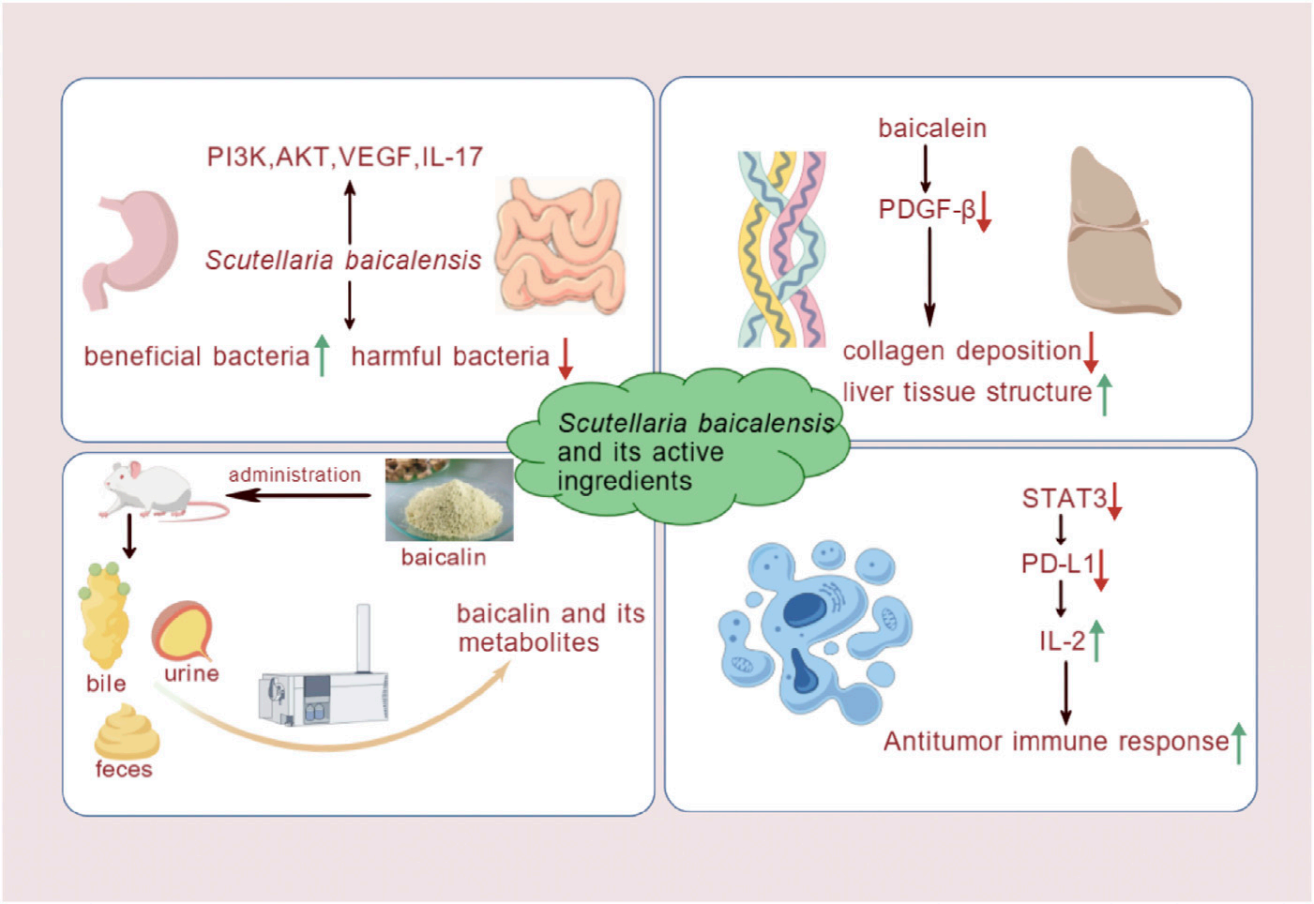
Baicalin had treated MAFLD by regulating signaling pathways and mechanisms involved in fatty acid metabolism, lipid synthesis and decomposition, insulin resistance, and gluconeogenesis (Created by BioGDP.com).

In summary, *S. baicalensis* has significantly improved glucose and lipid metabolism through the regulation of multiple signaling pathways and mechanisms, thereby effectively treating non-alcoholic fatty liver disease (Figure 4).

### 3.4 *S. baicalensis* treatment for MAFLD by other routes

*S. baicalensis* and its active metabolites have demonstrated multifaceted effects in the treatment of MAFLD. They not only modulated hepatic inflammation, hepatoprotection, and glucose-lipid metabolism but also exhibited potential mechanisms, including gut microbiota regulation, anti-hepatic fibrosis, and metabolic modulation. These studies have enriched the application of *S. baicalensis* in liver disease treatment and revealed its potential value. A comprehensive analysis of these studies provides a deeper understanding of the multifaceted roles of *S. baicalensis* and its active metabolites in the treatment of liver diseases.

The gut microbiota palys an important role in the pathogenesis of MAFLD. Hu et al. demonstrated that baicalin exerted its effects on the treatment of liver disease by regulating the gut-liver axis, improving gut barrier function, reducing intestinal permeability, and inhibiting inflammatory responses (Hu et al., 2021). Moreover, gut microbiota dysbiosis had been one of the key contributors to MAFLD. Studies have demonstrated that baicalin exerts inhibitory effects on a range of harmful bacteria. At a concentration of 500 μg/mL, baicalin significantly inhibits the biofilm formation of *Streptococcus* mutans by downregulating the expression of genes associated with biofilm formation and reducing the activity of related enzymes (Elango et al., 2021). Both *in vitro* and *in vivo*, baicalin exhibits notable antibacterial effects against *Pseudomonas aeruginosa*, inhibiting its biofilm formation and motility (Yan et al., 2024). Baicalin had regulates gut microbiota homeostasis by increasing the production of SCFAs, enhancing the abundance of beneficial bacteria such as Bifidobacterium and *Lactobacillus*, and inhibiting the growth of harmful bacteria. This improves gut microbiota balance and alleviated hepatic fat accumulation and inflammation (Ju et al., 2019). Dysbiosis of the gut microbiota may have facilitated the progression of MAFLD to liver fibrosis, hepatocellular carcinoma, and NASH. The mechanisms and pharmacological effects of gut microbiota modulation in MAFLD treatment are still in the early stages of research and further validation through cell and animal studies is required.

In MAFLD, liver fibrosis gradually develops due to the combined effects of metabolic disturbances, oxidative stress, and inflammatory responses. Liver fibrosis, a severe condition characterized by excessive accumulation of extracellular matrix, leads to impaired liver function. Under the stimulation of Wnt signaling caused by PyGO1 mutations, the replication of senescent hepatocytes is halted, activating ductal responses that exacerbate liver fibrosis in MAFLD patients. Consequently, liver fibrosis represents a critical factor in the progression of MAFLD (Fabris et al., 2024). Experimental studies have shown that *S. baicalensis* has shown significant effectiveness in the treatment of heart failure. Sun et al. used a CCl_4_-induced rat model of liver fibrosis and administered baicalein for a long period of time. The study found that baicalein inhibited the synthesis of PDGF-β receptor proteins, significantly reducing the degree of CCl_4_-induced liver fibrosis, as evidenced by decreased collagen deposition and improved liver tissue architecture (Sun et al., 2010). Taken together, *S. baicalensis* has likely ameliorates liver fibrosis, thereby potentially preventing the progression of MAFLD to more severe liver diseases, providing both theoretical and experimental evidence for the treatment of MAFLD.

HCC is one of the leading cancers worldwide, with over 600,000 deaths occurring annually due to HCC (Song et al., 2012). In the United States, the incidence of HCC has increased significantly due to MAFLD. Analysis of this study indicates that MAFLD was an important cause of HCC (Younossi et al., 2015). *S. baicalensis* and its metabolites have shown antitumor effects, although their mechanisms of interaction with molecular targets are still under investigation. Theoretically, their therapeutic potential in HCC may involve inhibiting cell proliferation, promoting apoptosis and autophagy, suppressing VEGF expression, and exerting anti-inflammatory effects (Ma et al., 2023). Ke et al. further validated through experiments that baicalin and baicalein inhibited STAT3 activity, downregulated IFN-γ-induced PD-L1 expression, restored T cell activity, and increased IL-2 secretion. These effects enhanced T cell-mediated antitumor immune responses, blocked the PD-L1/PD-1 signaling pathway, and strengthened antitumor immunity, thereby synergistically inhibiting tumor growth and improving the immune microenvironment (Ke et al., 2019).

Although flavonoids are generally not well-absorbed after being metabolized in the gut and liver, certain metabolites have shown significant pharmacological activity. As a metabolic disease, MAFLD may be modulated by baicalin and its aglycone through the regulation of metabolic processes related to the pathogenesis of MAFLD, providing new approaches for the treatment of metabolic disorders (Fang et al., 2020). Zhang et al. established a rat liver microsomal-hydrogel system and used the Phase II metabolism (MHCCS-II) cell culture system to predict the metabolic effects of drugs. They found that baicalein (BA) enhanced the antitumor effects on HepG2 and MCF-7 cells through metabolic processes. This study provided a potential framework for *in vitro* investigations into the pharmacological effects of baicalin metabolites, acting as substrates for UDP-glucuronosyltransferases (UGT) during Phase II metabolism (Zhang et al., 2019). Using ultra-performance liquid chromatography-tandem mass spectrometry (UPLC-MS/MS), the metabolism and excretion of *S*. *baicalensis* extract and its metabolites in rats were investigated. The results revealed that five metabolites—baicalin, baicalein, oroxylin A, oroxylin A-7-O-β-D-glucuronide, and scutellarin—were predominantly excreted in the urine, with lower amounts found in feces and bile. Baicalin, baicalein, and their metabolites were primarily eliminated via renal excretion (Zhou et al., 2024; Wang et al., 2012). However, variations in experimental animals, dosage, and detection methods may have produced different results in different studies.

**FIGURE 5:**
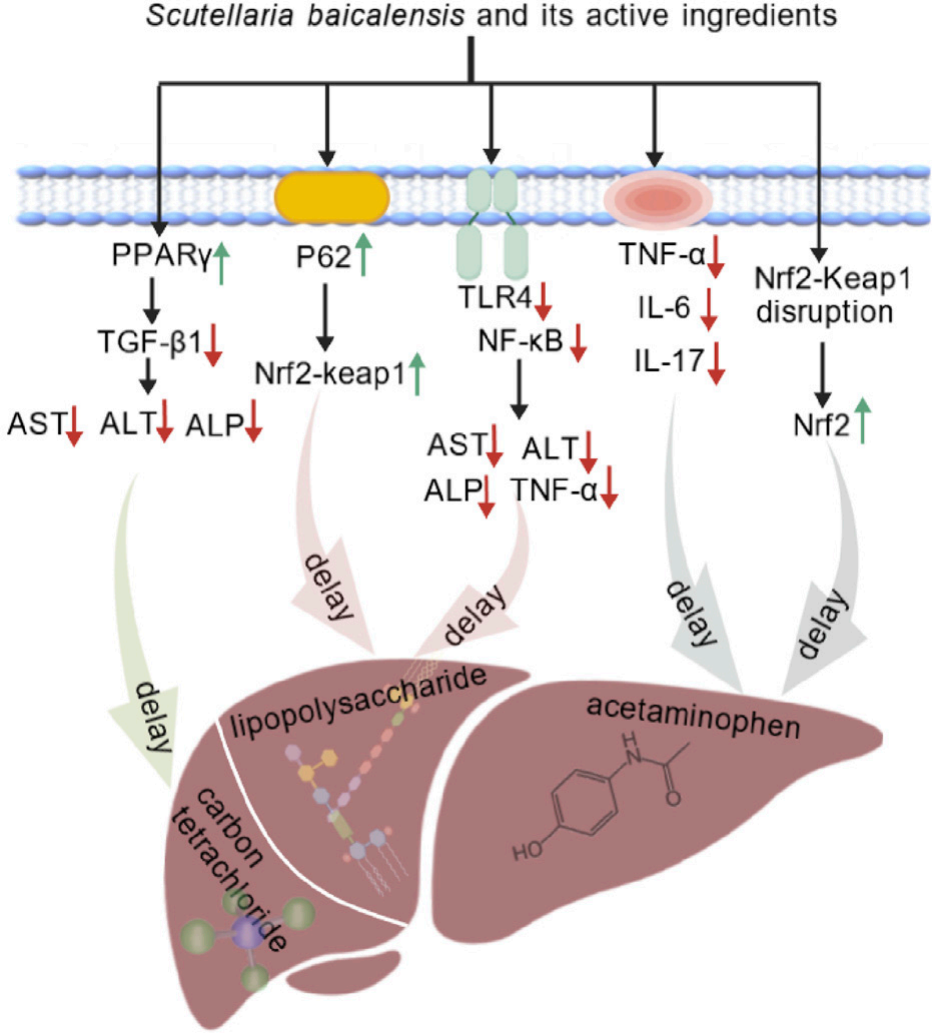
*S. baicalensis* and its active metabolites had provided theoretical and experimental evidence for the treatment of MAFLD by regulating intestinal microbiota, mitigating liver fibrosis, modulating metabolism, and elucidating potential mechanisms of action in liver cancer treatment (Created by BioGDP.com).

By reviewing the above literature, we have found that the use of *S. baicalensis* in the treatment of MAFLD provides strong scientific evidence. However, further studies are needed to deepen the understanding of its mechanisms of action and promote its clinical application (Figure 5).

## 4 *S. baicalensis* combined with other drugs for the treatment of MAFLD

The pathogenesis of MAFLD is complex, involving disturbances in lipid metabolism, inflammatory responses, and other factors. In recent years, progress has been made in studies exploring the combined use of *S. baicalensis* and other drugs in the treatment of MAFLD, providing new insights and methods for the comprehensive treatment of this disease.

Zhao et al. established an MAFLD model using HFDs and conducted experiments with different combinations of puerarin, baicalin, and berberine. The results indicated that the combined treatment of puerarin, baicalin, and berberine upregulated the expression levels of liver proliferator-activated receptor (PPAR)-γ and insulin receptor (IR). This contributed to improving insulin resistance as a therapeutic mechanism. Moreover, the combination therapy enhanced overall therapeutic efficacy through multiple targets (Zhao W. et al., 2016). Cui et al. investigated the therapeutic effects of *S. baicalensis* and Coptis chinensis rhizome on type 2 diabetes (T2DM) induced by a HFD combined with low-dose streptozotocin (STZ). The combination therapy primarily improved the pathological features of rats by regulating the expression of pro-inflammatory cytokines, key target proteins in the MAPK signaling pathway, insulin signaling, and the activity of enzymes related to glucose metabolism. The study also indicated that the combination treatment was more effective than monotherapy (Cui et al., 2018).

Liver fibrosis is a key intermediate step in the progression of MAFLD to more severe liver diseases, and its severity has influenced the prognosis and outcome of the disease. *S. baicalensis* has been found to play a therapeutic role in liver fibrosis, blocking the progression of MAFLD to more severe liver conditions. The combination of *S. baicalensis* and Rhei Rhizoma reduced liver fibrosis induced by dimethylnitrosamine (DMN), mainly by regulating redox status, inhibiting oxidative stress, and reducing the expression of α-smooth muscle actin (α-SMA), thereby protecting the liver from fibrotic damage (Pan et al., 2015).

With the increasing integration of Traditional Chinese Medicine (TCM) and Western medicine, not only has the compatibility of *S*. *baicalensis* with other Chinese herbal medicines attracted attention, but its combined use with modern pharmacological agents has also become a growing area of research interest. A study conducted on Otsuka Long Evans Tokushima Fatty (OLETF) rats demonstrated that co-administration of metformin with *S. baicalensis* resulted in more significant reductions in blood glucose and serum cholesterol levels compared to metformin alone, without adversely affecting the pharmacological actions of metformin (Han et al., 2017). In a 20-week clinical study conducted in 2016, the combination of *S. baicalensis* and metformin for the treatment of type 2 diabetes (T2D) was shown to improve glucose metabolism and significantly enhance glucose tolerance by modulating the composition and function of the gut microbiota, while also effectively reducing inflammatory markers (Shin et al., 2020).

Given the substantial overlap in pathophysiological mechanisms between MAFLD and T2D—particularly the tight association between gut microbiota dysbiosis and metabolic inflammation—this combined therapeutic strategy, which targets the gut microbiota to improve metabolic and inflammatory status, may offer a novel approach and theoretical basis for clinical interventions in MAFLD.

**FIGURE 6:**
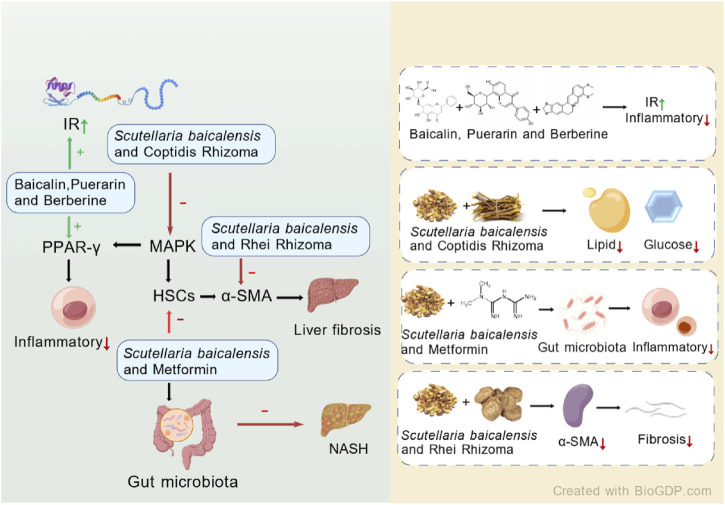
Potential mechanisms of *S. baicalensis* combined with other drugs in the treatment of MAFLD (Created by BioGDP.com).

Figure 6 summarizes the potential mechanisms of *S. baicalensis* combined with other drugs in the treatment of MAFLD. The combination of *S. baicalensis*, puerarin, and berberine has been confirmed to upregulate the expression of PPAR-γ and IR in the liver, thereby enhancing insulin receptor sensitivity and reducing the generation of inflammatory cytokines. The combined application of *S. baicalensis* and Coptis chinensis can inhibit the expression of P38, ERK, and JNK proteins in the MAPK signaling pathway, while upregulating the levels of key glycolytic enzymes such as GK, PFK, PK, and GS, thus accelerating glycolytic reactions and fatty acid synthesis, and reducing glucose and lipid levels in the liver. Moreover, the combination of *S. baicalensis* and metformin can modulate the gut microbiota, thereby improving glucose metabolism and reducing inflammation. These effects subsequently impact the development of MAFLD, effectively treating the condition and impeding its progression. Additionally, the combination of *S. baicalensis* and rhubarb can alleviate liver fibrosis by reducing the expression of the important marker α-SMA in activated HSCs. In summary, the combination of *S. baicalensis* with other drugs has shown broad application prospects.

## 5 *S. baicalensis*-pharmacokinetics, bioavailability, and toxicity

Given the significant pharmacological effects of baicalein in the treatment of MAFLD, its pharmacokinetics had become a key focus of our attention. Numerous studies in the literature have also explored this topic. However, the low oral bioavailability of baicalin (<2.2%) and its poor solubility may limit its clinical efficacy (Lu et al., 2022). The reasons may be as follows: *S*. *baicalensis* and its major metabolites, such as flavonoid glycosides, are highly polar compounds that cannot pass through the lipid bilayer via passive diffusion, resulting in poor absorption in the intestine (Fang et al., 2017). Baicalin is the predominant metabolite found in blood following oral administration of baicalin and baicalein. Studies indicate that baicalin undergoes a complex metabolic process *in vivo*, which includes gastrointestinal hydrolysis, enterohepatic circulation, and carrier-mediated transport. Baicalin is primarily metabolized via glucuronidation, and it is hydrolyzed in the gastrointestinal tract by microbial enzymes into its active metabolite, baicalein (Xiang et al., 2021). In the liver and intestine, baicalein is predominantly glucuronidated by UDP-glucuronosyltransferases (UGTs), resulting in the formation of baicalin and baicalein-6-O-glucuronide as two major metabolites (Son et al., 2021). Studies investigating the pharmacokinetics of orally administered baicalin, influenced by gut microbiota, and the concurrent role of hepatic enzymes and transport proteins in baicalein metabolism, suggest that baicalin and its metabolites are primarily excreted via bile, with a portion being eliminated in urine (Kang et al., 2014; Zhang et al., 2011).

Although the oral bioavailability of baicalin is relatively low, studies have demonstrated that its tissue distribution is predominantly concentrated in the liver, kidneys, and lungs. After 30 minutes of oral administration of baicalin liposomes, the drug concentrations in the liver, kidneys, and lungs were 5.59, 2.33, and 1.25 times higher, respectively, compared to the baicalin suspension (Wei et al., 2014). Moreover, studies have shown that the drug concentration in the lungs after intravenous injection was significantly higher than in the liver, kidney, spleen, and other tissues as well as in plasma (Zhao Q. et al., 2016). Moreover, in rats with middle cerebral artery occlusion (MCAO), the baicalin concentration in lung tissue was higher than that in the kidneys or liver after oral administration (Zhu et al., 2013). Other studies have found that the highest concentration of baicalin was reached in the kidneys after oral administration, whereas the compound accumulated predominantly in the lungs after intravenous injection of liposomal baicalin (Zhao Q. et al., 2016).

To improve the efficacy and address the limited bioavailability of single-agent drugs, drug complexes, nanoparticles, nanoliposomes, and other synergistic compound delivery methods have been employed. Numerous studies have shown that liposomal carriers could enhance the oral bioavailability of drugs (Liu et al., 2020; Wei et al., 2014). Targeted nanoparticles promote the *in vivo* and *in vitro* bioavailability of baicalin. In cells treated with baicalin liposomes (BAA1), ApoA1-modified nanoparticles effectively enhanced baicalin uptake in HEPG-2 cells (Xu et al., 2022). An innovative oral baicalein-loaded solid lipid nanoparticle (SLNB) significantly enhanced the bioavailability of baicalein. Compared to free baicalein, the oral relative bioavailability of SLNB increased by approximately 300% (Joshi et al., 2021). Xxx et al. administered baicalin liposomes nasally for the treatment of cerebral ischemia-reperfusion injury in rats. The results demonstrated that baicalin liposomes exhibited favorable pharmacokinetic properties *in vivo*, effectively enhancing the concentration of the drug in brain tissue, while showing good pharmacological efficacy and safety (Xiang et al., 2020). Zhao et al. prepared baicalin-loaded nanoemulsions (BAN-1 and BAN-2) through internal or external drug addition methods and evaluated them both *in vitro* and *in vivo*. Their findings indicated that the baicalin-loaded nanoemulsions, especially BAN-1, were highly effective in improving the oral bioavailability of baicalin (Zhao et al., 2013). Furthermore, the use of novel formulations, such as MSCs-derived exosomes, has the potential to improve bioavailability (Zhao et al., 2022).

The metabolism of *S. baicalensis* and its active metabolites has been shown to influence their activity and toxicity. An oxidative stress study on non-alcoholic fatty liver disease (MAFLD) demonstrated, through CCK-8 assays, that baicalin concentrations ranging from 0.01 nM to 100 µM had no cytotoxic effects on HepG2 cells at 24 and 48 h (Cheng et al., 2017). A dose of 400 mg·kg^−1^·d^−1^ of baicalin was administered for 14 weeks to high-fat diet mice without toxicity, significantly improving insulin resistance and lipid abnormalities in skeletal muscle. Clinical evaluations of a single oral dose of 100–2,800 mg baicalein in healthy subjects have shown good tolerance with no evidence of liver or kidney toxicity (Li et al., 2014). However, currently, there are no clinical research literatures reporting on the potential risks of long-term use of *S. baicalensis* and its contraindications. While current research has demonstrated the short - term safety and efficacy of *S*. *baicalensis*, future studies should prioritize long - term clinical trials and translational research to define its potential risks, contraindications, and optimal use in treating MAFLD.

## 6 Production development

**FIGURE 7:**
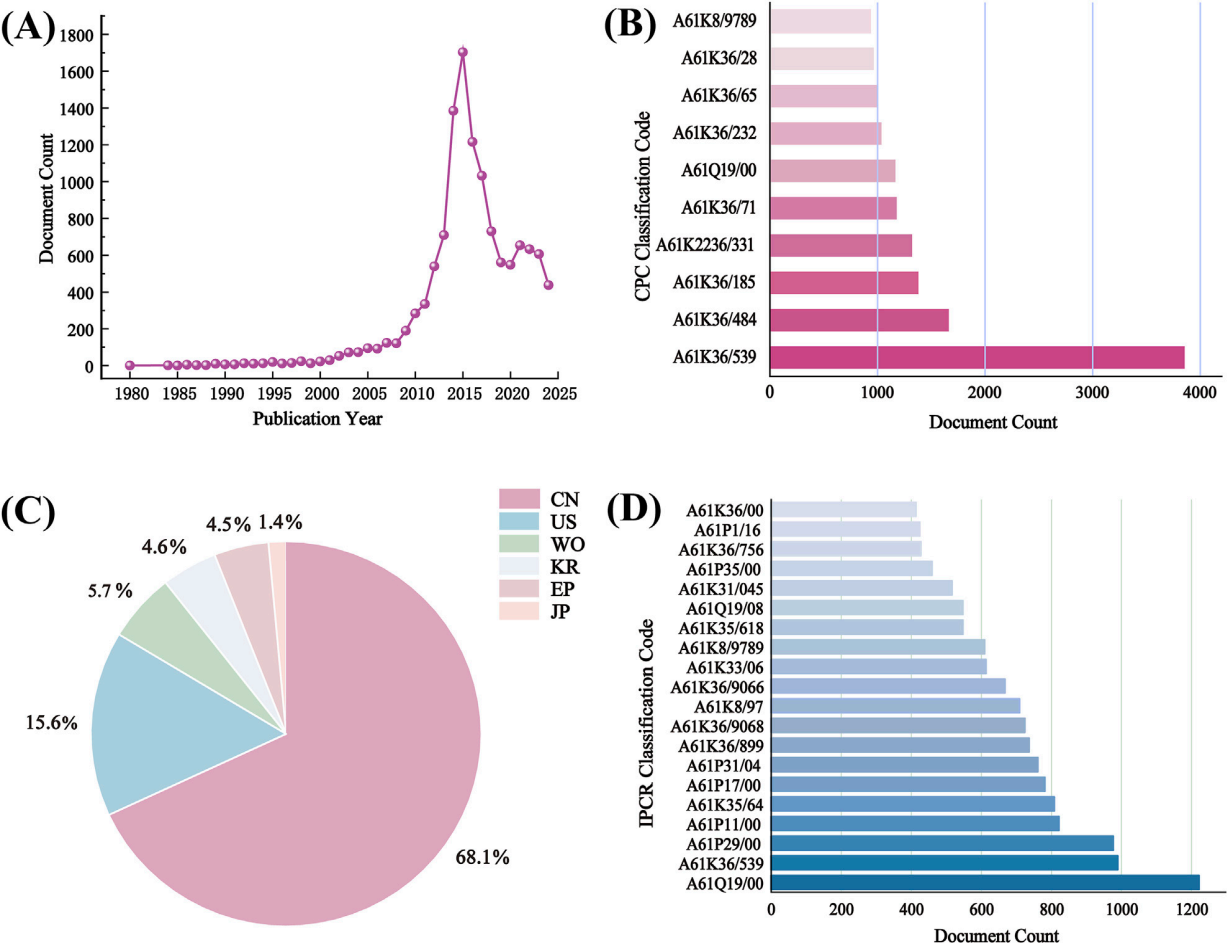
Analysis of 12403 patents browsed on LENS.ORG (https://www.lens.org/) using search terms “*S. baicalensis*”. **(A)** Patent applications per year; **(B)** CPC classification code; **(C)** Jurisdictions (CN, China; US, United States; WO, World Intellectual property organization; KR, South Korea; EP, European Patent Office; JP, Japan); **(D)** IPCR classification code.

*S. baicalensis*, a traditional Chinese medicine with a rich history, has garnered significant attention due to its remarkable pharmacological effects. It is extensively utilized in traditional Chinese medicine for its abilities to clear heat and detoxify, reduce inflammation, and protect the liver. Recent modern research has further validated its considerable potential in antibacterial, antiviral, and anti-tumor applications. Currently, the total number of AR patents worldwide has reached 12,403, with China holding the majority, accounting for over three-fifths of the total. Detailed information regarding AR patents is presented in Figure 7. In the 2020 edition of the Chinese Pharmacopoeia, the Traditional Chinese Medicine (TCM) formulas based on *S. baicalensis* include Qinian Pian, Jingtaihong Zhike Granules, Xinqin Pian, Zhaoqin Qingre Heji, Compound Qinlan Oral Liquid, and Gegen Qinlian Wan. Additionally, the 2020 edition of the Chinese Pharmacopoeia includes a total of 248 formulas containing *S. baicalensis*, involving 185 prescriptions. The main dosage forms are as follows: pills (31.05%), tablets (18.95%), capsules (15.32%), granules (14.11%), and oral liquids (10.89%).

The market demand for *S. baicalensis* has been increasing year by year, with widespread applications in the fields of pharmaceuticals, health supplements, and cosmetics. The China Food and Drug Administration (CFDA) (nmpa. gov. cn) has announced 82 *S. baicalensis* related drugs, including Huangqin tablets, Huangqin capsules, Baicalin capsules, Xiongdan Huangqin eye drops, Sanhuang tablets, Yinzhihuang oral solution, etc.

In modern society, the fast-paced lifestyle and increasing work pressures have led many people to gradually overlook the importance of healthy eating and regular exercise. This unhealthy lifestyle has evident impacts on the body, with gastrointestinal issues becoming common among many individuals. To address this, *S. baicalensis* sesame oil soft capsules have emerged as a specialized health product aimed at improving gastrointestinal health.

With the rapid development of the economy, people’s awareness of skincare has also been continuously rising. Simultaneously, there is a growing preference for natural and harmless cosmetics, leading to the widespread incorporation of natural plant extracts in products. Plant extracts are commonly used in cosmetics, and *S. baicalensis*, as a traditional Chinese medicinal herb, has found extensive applications in this field. In 2020, the number of recorded extracts from *S. baicalensis* roots exceeded 20,000. Among the recorded metabolites primarily focused on soothing and anti-aging properties, *S. baicalensis* root extract ranked among the top ten in both quantity and growth rate. Currently, *S. baicalensis* is mainly applied in cosmetics for purposes such as whitening, soothing, and sun protection. To better utilize *S. baicalensis* resources, it is essential to conduct in-depth, multifaceted research on its mechanisms of action, exploring biochemical, cellular, animal, and human perspectives. Additionally, as consumer demand for anti-aging products increases, it is anticipated that *S. baicalensis* will have broad application prospects in this area.

Moreover, with the advancement of modern pharmacological research, the therapeutic potential of *S*. *baicalensis* in liver injury and liver diseases has garnered increasing attention. *S*. *baicalensis* and its primary active metabolites are widely incorporated in various TCMs for the treatment of liver damage and liver diseases. For instance, TCM formulations listed in the Chinese Pharmacopoeia, such as Longdan Xiegan Wan and Qinggan Lidan Oral Liquid, contain *S*. *baicalensis* and are used for clearing heat, detoxification, liver protection, and bile regulation. Additionally, Shugan Ning Injection, composed of *S*. *baicalensis* extract among other metabolites, is used as an adjunctive therapy for viral hepatitis and liver dysfunction. Furthermore, commercially available Baicalin capsules are commonly employed as supplementary treatments for viral hepatitis and liver dysfunction, exerting hepatoprotective effects through multiple mechanisms, including antioxidant, anti-inflammatory, and antiviral actions. Some compound preparations, such as Chaiqin Qingning Capsules, combine various herbal metabolites and are used to alleviate symptoms of colds and fever while improving liver function. To further expand the therapeutic applications of *S*. *baicalensis* in liver diseases, it is essential to delve deeper into its pharmacological mechanisms and safety profiles at the cellular, animal model, and clinical levels, facilitating its transition from a traditional herbal remedy to a modern pharmaceutical agent.

Furthermore, *S. baicalensis* has a long history of application in dietary practices, particularly in nourishing diets aimed at enhancing health. This botanical drug can be easily integrated into various culinary creations, seamlessly incorporating health benefits into daily meals. It pairs well with a variety of metabolites, including meats, rice, and vegetables, allowing for the preparation of delicious and nutritious dishes, such as red date and Huangqin chicken stew and Huangqin stewed black beans. The recipe for Huangqin chicken stew, found in the “Medicinal Cuisine Handbook,” is a nourishing dish suitable for those suffering from Qi and blood deficiency due to serious illness, prolonged sickness, or excessive postpartum blood loss. *S. baicalensis* is known for its ability to tonify Qi, elevate Yang, strengthen the surface, stop sweating, promote diuresis, nourish blood, expel toxins, and aid in wound healing. The hen used in the dish is beneficial for invigorating Qi and nourishing blood, enhancing overall vitality. Together, they provide a synergistic effect that nourishes Qi, replenishes blood, and strengthens the essence. These traditional recipes not only highlight the versatility of this botanical drug but also emphasize its role in promoting overall health.

Despite the optimistic outlook, challenges remain between foundational research and the industrialization of products centered around *S. baicalensis*. Ongoing research aimed at revealing active metabolites and understanding their mechanisms of action is crucial for overcoming these obstacles. As research progresses, it is expected that effective and safe clinical drugs and health products will soon be developed, further expanding the application prospects of *S. baicalensis*, not only in the realms of food and dietary supplements but also in everyday essentials. Ultimately, this will contribute to the establishment of a healthier society. Through continuous innovation and exploration, the full potential of *S. baicalensis* will be realized, benefiting individuals and communities worldwide.

## 7 Discussion and outlook

There are currently no internationally approved treatments for MAFLD. The causes are diverse, the cost of screening is high, and the results remain uncertain. No satisfactory preventive or therapeutic medications have been identified (Liu et al., 2023; Younossi et al., 2018; Qu et al., 2021). Interventions based solely on lifestyle modifications have led to limited improvement in MAFLD, and in cases of severe disease, they have failed to prevent further progression. Therefore, research into effective treatments for MAFLD is of great importance.

As shown in Table 1, this review summarizes a total of 57 experimental studies involving various hepatic and metabolic diseases, including fatty liver disease, liver cirrhosis, hepatitis, and diabetes. Among these, seven studies involved *in vitro* experiments, primarily utilizing human hepatocellular carcinoma cell lines such as HepG2 and SMMC-7721, as well as engineered liver models. These studies mainly focused on lipid metabolism and inflammatory pathways, such as AMPK/NF-κB signaling. However, these models lack a complete hepatic microenvironment, including immune interactions and dynamic fibrogenesis, which limits their ability to fully recapitulate the complex pathophysiological features of MAFLD.

**TABLE 1:** Mechanisms and targets of *S. baicalensis* in treating MAFLD and related diseases: A summary of experimental studies.

Disease	Experimental model	Dose	Duration	Positive control	Targets/pathways/mechanisms	Effects	Refs
MAFLD	Male SD rats	Huangqin decoction800 mg/kg/day	8 weeks	Polyene lecithin choline	TLR4/NF-κB/NLRP3	TLR4/NF-κB↓	Yan et al. (2023)
Liver cirrhosis	Adult male SD rats	Baicalin25/75 mg/kg/day	4 weeks	thioacetamide	TGF-β1/NOX4/NF-κB/NLRP3	NLRP3/Caspase-1↓	Zaghloul et al. (2022)
MAFLD	Male C57BL/6J mice at 8 weeks	Baicalin 100/200/400 mg/kg/day	14 weeks	-	SREBP1/Nrf2/NF-κB	TG/TC/LDL↓, HDL↑	Gao et al. (2023)
HLH	C57BL/6J mice at 6–8 weeks	Baicalin 200 mg/kg/day	3 days	-	Kupffer PANoptotic	TNF-α/IFN-γ↓	You et al. (2024)
MAFLD	Male C57BL/6J mice at 6–8 weeks	Baicalin 50 mg/kg/day	4 weeks	-	TLR4	ALT/AST/p-p38/p-p65↓	Liu et al. (2020)
Obese	Male C57BL/6 mice at 6 weeks	Baicalin 200/400 mg/kg/day	8 weeks	-	TNF-α, CCL2, F4/80	Ly6C^hi^/M1/ATM/M1/Kupffer↓, M2 ATM/CD4+ T cell↑	Noh et al. (2021)
Liver injury	ICR mice at 6 weeks	Baicalin 100 mg/kg/day	3 days	-	NF-κB	MCP-1/IL-6/ROS/TNF-α↓	Park et al. (2017)
NASH	Male C57BL/6J mice at 7–8 weeks	Baicalin 0.5% w/w	12 weeks	-	JNK	GSH/SOD/HDL↑, MDA/ALT/AST/LDL↓	Zhong and Liu (2018)
NASH	Male SD rats at 10 weeks	Baicalein 10 mg/kg/day	8 weeks	-	Nrf2/HO-1	SOD↑,TNF-α/IL-6↓	Xin et al. (2014)
MAFLD	Male db/m mice and db/db mice	Baicalin 50/100/200 mg/kg/day	4 weeks	metformin	p62–Keap1–Nrf2	HO-1/GCLC/SOD/T-AOC/GSH/CAT↑, MDA↓	Liu et al. (2023)
MAFLD	Tissue-Engineered liver	Baicalin 100 µM	-	-	ROS	SOD/GSH↑, ROS/MDA↓	Gao et al. (2022)
Acute Liver Injury	C57BL/6 mice at 6–8 weeks	Baicalin 150 μg/mice	-	-	P62 - Keap1 - NRF2	P62/Keap1/NRF2↓	Zhao et al. (2022)
Acute Liver Injury	Male C57BL/6 mice at 8 weeks	Baicalin 30 mg/kg	-	-	IL-17	TNF-α/IL-6/IL-17/MPO↓	Liao et al. (2016)
Acute Liver Injury	Male C57BL/6 mice	Baicalein/Baicalin 40/80 mg/kg/day	7 days	-	Keap1-Nrf2	ROS/Keap1/Nrf2↓	Shi et al. (2018)
NASH	Male SD rats at 2–3 weeks	Baicalin magnesium 50/150 mg/kg/day	2 weeks	-	NLRP3/Caspase - 1/IL - 1β	NLRP3/Caspase-1/IL-1β↓	Guan et al. (2023)
Liver Inflammation	1-day-old Beijing white chickens	Baicalin 50/100/200 mg/kg	-	-	TLR4 -NF - κB	TLR4/NF-κB↓	Cheng et al. (2017)
Liver injury	Male SD rats	Baicalin 25/50/100 mg/kg	1 week	silymarin (200 mg/kg)	TGFβ1, PPARγ	PPARγ↑,ALT/AST/ALP/tgf-β1↓	Qiao et al. (2011)
MAFLD	Male SD rats	Baicalein 25,100 mg/kg			SPEBP1C/ChREBP, AMPK/PPARα	SPEBP1C/ChREBP↓ AMPK/PPAα↑	Li et al. (2022a)
hepatic steatosis	DIO mice	Baicalin 400 mg/kg			CPT1A	CPT1A↑	Dai et al. (2018)
ALD	Male 6-week-old SD rats	Baicalin 200 mg/kg			PNPLA3 ATGL	PNPLA3-ATGL↓	Li et al. (2022b)
MAFLD	C57BL/6J mice	Baicalin 100 mg/kg			SREBP1/Nrf2/NF-κB	SREBP1 ↓ NF-κB↓ Nrf2 ↑	Gao et al. (2022)
MAFLD	KK-Ay mice	Baicalin 12.5,25,50 mg/kg			AMPK SREBP	AMPK↑ SREBP-1c↓	Chen et al. (2018)
MAFLD	Male SD rats. human hepatoma HepG2 cells	Baicalin 80 mg/kg, 5 and 10 µmmol/L			P-AMPK,P-ACC SREBP-1c,AMPK	P-AMPK/P-ACC↑ SREBP-1c↓ AMPKα↑	Guo et al. (2009)
Diabetes	human hepatoma HepG-2 cell	Baicalin 0–50 mM			AMPK,AKT	AMPK↑ AKT↑	Wang et al. (2017)
Diabetes	Insulin-resistant-HepG2 cell Male C57BL/6J mice	Baicalin 20/50 µM 50 and 100 mg/kg			IRS/PI3K/AKT,GLUT4,GSK-3β	IRS/PI3K/AKT↓ GLUT4↓ GSK-3β↑	Miao et al. (2024)
Insulin resistance	Male C57BL/6J mice	Baicalin 100,200 and 400 mg/kg			AMPK/ACC AKT/GSK-3β	AMPK/P-ACC↑ AKT↑ GSK-3β↓	XI et al. (2016)
Diabetes	Male C57BL/6Jmice	Baicalein 50 mg/kg			p38MAPK PGC-1α	p38MAPK↓ PGC-1α↓	Fang et al. (2019)
T2D	Male C57BL/6 J mice at 8 weeks old	Baicalin 200 mg/kg			SCFAs	SCFAs↑	Ju et al. (2019)
HF	The SPF grade SD male rats	Baicalin 25 mg/kg			PI3K/AKT、IL-17 VEGF	PI3K/AKT↓ IL-17↓ VEGF↓	Liu et al. (2023)
HF	Male SD rats	Baicalein 20, 40, or 80 mg/kg			PDGF-β	PDGF-β↓	Sun et al. (2010)
HCC	SMMC-7721 cells and HepG2 cell	Baicalein and baicalin 10 μM and 40 μM			STAT3 PD-L1	STAT3↓ PD-L1↓	Ke et al. (2019)
MAFLD	Male SD rats	Baicalein 25, 100 mg/kg	5 weeks		SPEBP1C/ChREBP, AMPK/PPARα	SPEBP1C/ChREBP↓ AMPK/PPAα↑	Li et al. (2022a)
hepatic steatosis	DIO mice	Baicalin 400 mg/kg	12 weeks		CPT1A	CPT1A↑	Dai et al. (2018)
ALD	Male 6-week-old SD rats	Baicalin 200 mg/kg	4 weeks		PNPLA3、ATGL	PNPLA3-ATGL↓	Li et al. (2022b)
MAFLD	C57BL/6J mice	Baicailin 100 mg/kg	24 weeks		SREBP1/Nrf2/NF-κB	SREBP1 ↓ NF-κB↓Nrf2 ↑	Gao et al. (2022)
MAFLD	KK-Ay mice	Baicalin 12.5,25,50 mg/kg	18 days		AMPK/SREBP	AMPK↑ SREBP-1c↓	Chen et al. (2018)
MAFLD	Male SD rats. Human hepatoma HepG2 cells	Baicalin baicalin 80 mg/kg. 5and10 μmmol/L	16 weeks		P-AMPK,P-ACC,SREBP-1c,AMPK	P-AMPK↑P-ACC↑ SREBP-1c↓ AMPKα↑	Guo et al. (2009)
Diabetes	human hepatoma HepG-2 cells	Baicalin 0–50 mM	-		AMPK/AKT	AMPK↑ AKT↑	Wang et al. (2017)
insulin resistance	Male C57BL/6J mice	Baicalin 100, 200, and400 mg/kg	14 weeks		AMPK/ACC Akt/GSK-3β	AMPK/P-ACC↑ Akt↑GSK-3β↓	XI et al. (2016)
Diabetes	Male C57BL/6Jmice	Baicalein 50 mg/kg	21 days	metformin	p38MAPK/PGC-1α	p38MAPK↓ PGC-1α↓	Fang et al. (2019)
T2D	Male C57BL^−/−^6 J mice (8 weeks old)	Baicalin 200 mg/kg	-	-	SCFAs	SCFAs↑	Ju et al. (2019)
HF	The SPF grade SD male rats	Baicalin 25 mg/kg	8 weeks		PI3K/AkT、IL-17 VEGF	PI3K/AkT↓IL-17↓ VEGF↓	Liu et al. (2023)
HF	male SD rats	Baicalein 20, 40, or 80 mg/kg	10 weeks		PDGF-β	PDGF-β↓	Sun et al. (2010)
HCC	SMMC-7721 cells and HepG2 cell	Baicalein and baicalin 10 μM or40 μM	24 h		STAT3 PD-L1	STAT3↓ PD-L1↓	Ke et al. (2019)
MAFLD	adult male Sprague-Dawley rats	Baicalin and Berberine 100 mg/kg	8 weeks	Rosiglitazone	PPAR-γ, IR	ALT/AST/TC/TG/LDL↓,HDL/TNF-α/IL-6↑	Zhao W. et al. (2016)
Liver fibrosis	6-week-old male Wistar rats	SRE 1.25/6.25 mg/kg	3 weeks	-	ROS	ROS/α-SMA↓	Pan et al. (2015)
T2DM	SD rats	crude herbs 6.3 g/kg	1 month	metformin	MAPK	P38/ERK/JNK↓,GK/PFK/PK/GS↑	Cui et al. (2018)
T2D	OLETF rats	S. baicalensis extract (200 mg/5 mL/kg/day)	12 weeks	metformin	CYP7A1/NR1H4	LDLR↑ HMGCR↓	Han et al. (2017)

In contrast, 50 studies employed *in vivo* models, predominantly using SD rats (29 studies) and C57BL/6 mice (18 studies). The disease models included alcoholic liver disease (ALD); short-term ethanol administration for 4 weeks in SD rats, without the use of classical chronic alcohol consumption models), NAFLD/MAFLD or NASH; induced by high-fat diet or in genetic db/db mice, and liver fibrosis (induced by thioacetamide, TAA). Notably, the ALD models used in these studies feature short intervention durations and simplified pathogenic mechanisms, which differ significantly from the chronic disease course observed in human alcoholism.

It is worth noting that the vast majority of these studies are preclinical, with only one clinical trial reported to date. There remains a lack of robust and consistent clinical data to support the findings. Future research should focus on conducting multicenter, long-term clinical trials (lasting 6 months to 2 years) with large sample sizes encompassing various stages of disease progression. These studies should integrate biochemical markers (e.g., liver enzymes), imaging techniques, and histopathological evaluation to comprehensively assess drug efficacy and safety. Bridging this translational gap between basic research and clinical application remains a critical priority.

*S. baicalensis*, as a promising therapeutic agent, has been extensively studied for its efficacy in treating MAFLD. A substantial body of research has demonstrated its diverse therapeutic activities, including anti-inflammatory, antioxidant, metabolic regulation, and hepatoprotective properties, which provide hope for the treatment of MAFLD patients. However, significant challenges remained in translating *S. baicalensis* into clinical therapy for MAFLD.

At present, most clinical studies and animal experiments related to *S. baicalensis* focused primarily on adult animals and adult populations. However, the incidence of MAFLD in children and adolescents is steadily increasing and has become one of the most important pathogenic factors of chronic liver disease in children (Hatton et al., 2018). It is still unclear whether there are differences in the pathophysiological mechanisms of MAFLD between these two age groups (Nobili et al., 2016). Additionally, there are significant differences in drug sensitivity between children and adults (Assunção et al., 2017). Therefore, developing effective treatment strategies for children suffering from this disease holds great value.

(a) During the drug development process, the active metabolites of *S. baicalensis*, due to their complexity and diversity, along with the challenges in standardizing quality, led to instability in therapeutic effects. Therefore, ensuring the quality and stability of *S. baicalensis* materials has become one of the key directions for future research.

In addition, the bioavailability of the active metabolites of *S. baicalensis* was relatively low and the absorption in the gastrointestinal tract was limited after oral administration, which significantly affected the therapeutic efficacy. Pharmacokinetic studies of *S. baicalensis* and its active metabolites revealed that the drug was primarily concentrated in the lungs and kidneys after intravenous injection of baicalin. However, in the context of *treating MAFLD with S. baicalensis*, the goal was to target the liver. Therefore, the development of new formulations of *S. baicalensis* and its active metabolites was necessary to enable targeted delivery to the liver while improving the bioavailability of baicalin. This represented one of the major challenges for future research on *S. baicalensis* and its active metabolites in the treatment of MAFLD.

(b) Although *S. baicalensis* had demonstrated multi-target potential in the treatment of MAFLD, the underlying mechanisms were not yet fully understood. The multi-target mechanisms of *S. baicalensis* were intertwined with the complex pathophysiology of MAFLD, making research more difficult. When designing and evaluating studies on *S. baicalensis* for the treatment of MAFLD, these influencing factors had to be carefully taken into account, and targeted research strategies had to be developed. To date, research on *S. baicalensis* in MAFLD has primarily focused on animal models and small-scale preclinical studies, with a lack of large-scale, multi-center, randomized controlled clinical trials to validate its efficacy and safety in diverse populations.
(c) Existing studies have provided limited insight into the long-term efficacy and potential adverse effects of *S. baicalensis* in the treatment of MAFLD. It remained to be determined whether prolonged use of *S. baicalensis* could affect liver and kidney function or whether significant drug interactions or other side effects had occurred. Further systematic studies were required to ensure safety and feasibility for clinical application.

In conclusion, *S. baicalensis* and its active compounds have demonstrated complex and diverse pharmacological effects that may have had significant implications for the treatment of MAFLD. Mechanisms of action included inhibiting liver inflammation, providing liver protection, and regulating glucose and lipid metabolism. Additionally, *S. baicalensis* alleviated hepatic fat accumulation by modulating gut microbiota homeostasis. Furthermore, *S. baicalensis* alleviated fat accumulation in the liver by modulating the homeostasis of the gut microbiota. In addition, it has shown a synergistic effect in combination with other drugs, offering new possibilities for personalized treatment. These results suggest that *S. baicalensis* has significant potential as a drug candidate for the treatment of MAFLD. However, in order for *S. baicalensis* to be used in the clinical treatment of MAFLD, further research was required to ensure the stability of its quality, investigate the liver-targeting effects of the experimental drug, improve its bioavailability, and explore the underlying mechanism.

Despite existing evidence suggesting that *S. baicalensis* and its major active metabolites, such as baicalin and baicalein, hold therapeutic potential in the management of MAFLD, several limitations remain. Most studies to date have been conducted primarily in animal models, including high-fat diet and alcohol-induced models, with heterogeneous methodologies that undermine the comparability of findings and their clinical translational value. Furthermore, systematic studies on the pharmacokinetic properties, bioavailability, and liver-targeting distribution characteristics of *S. baicalensis* metabolites are still lacking, particularly with regard to their metabolic transformation mechanisms in relation to gut microbiota. More importantly, clinical investigations focusing on the use of *S. baicalensis* for the treatment of MAFLD are limited, and robust, high-quality clinical evidence supporting its safety and efficacy is still absent.

Future research should focus on a more in-depth understanding of the *in vivo* metabolism of key metabolites of *S. baicalensis*, elucidating their mechanisms of action. Concurrently, the development of novel drug delivery systems, including nanoparticles, liposomes, and prodrugs, is essential to enhance their bioavailability and liver targeting. Further optimization of animal models to better reflect human metabolic characteristics, along with strengthened preclinical toxicology studies, will facilitate the translation of these findings into clinical applications. Additionally, exploring the interactions between *S. baicalensis*, dietary factors, gut microbiota, and host metabolic networks could provide valuable insights for personalized intervention strategies. Overall, continued efforts in both fundamental mechanistic research and formulation optimization, alongside the gradual implementation of systematic clinical trials, are necessary to lay a solid foundation for the application of *S. baicalensis* in the prevention and treatment of MAFLD.
